# Prevalence and sociodemographic correlates of meeting the Canadian 24-hour movement guidelines among latin american adults: a multi-national cross-sectional study

**DOI:** 10.1186/s12889-022-12613-2

**Published:** 2022-02-03

**Authors:** Gerson Ferrari, Claudia Alberico, Clemens Drenowatz, Irina Kovalskys, Georgina Gómez, Attilio Rigotti, Lilia Yadira Cortés, Martha Yépez García, Maria Reyna Liria-Domínguez, Marianella Herrera-Cuenca, Miguel Peralta, Adilson Marques, Priscila Marconcin, Carlos Cristi-Montero, Ana Carolina B. Leme, Ioná Zalcman Zimberg, Claudio Farías-Valenzuela, Mauro Fisberg, Scott Rollo

**Affiliations:** 1 Universidad de Santiago de Chile (USACH), Escuela de Ciencias de la Actividad Física, el Deporte y la Salud, Santiago, Chile; 2 Laboratorio de Rendimiento Humano, Grupo de Estudio en Educación, Actividad Física y Salud (GEEAFyS), Universidad Católica del Maule, Talca, Chile; 3 JLC Biomedical and Biotechnology Research Institute (BBRI), North Carolina Central University, 1801 Fayetteville St, 27707 Durham, NC USA; 4 Division of Sport, Physical Activity and Health, University of Education Upper Austria, 4020 Linz, Austria; 5 Carrera de Nutrición, Facultad de Ciencias Médicas, Pontificia Universidad Católica Argentina, Buenos Aires, Argentina; 6 Departamento de Bioquímica, Escuela de Medicina, Universidad de Costa Rica, San José, Costa Rica; 7 Centro de Nutrición Molecular y Enfermedades Crónicas, Departamento de Nutrición, Diabetes y Metabolismo, Escuela de Medicina, Pontificia Universidad Católica, Santiago, Chile; 8 Departamento de Nutrición y Bioquímica, Pontificia Universidad Javeriana, Bogotá, Colombia; 9 Colégio de Ciencias de la Salud, Universidad San Francisco de Quito, Quito, Ecuador; 10 Instituto de Investigación Nutricional, La Molina, Lima, Peru; 11 Faculty of Health Sciences, Universidad Peruana de Ciencias Aplicadas (UPC), Lima, Peru; 12 Centro de Estudios del Desarrollo, Universidad Central de Venezuela (CENDES- UCV)/Fundación Bengoa, Caracas, Venezuela; 13 Faculdade de Motricidade Humana, CIPER, Universidade de Lisboa, Lisbon, Portugal; 14 Faculdade de Medicina, ISAMB, Universidade de Lisboa, Lisbon, Portugal; 15 KinesioLab, Research Unit in Human Movement Analysis, Instituto Piaget, Almada, Portugal; 16 Physical Education School, IRyS Group, Pontificia Universidad Catolica de Valparaiso, Valparaiso, Chile; 17 Centro de Excelencia em Nutrição e Dificuldades Alimentaes (CENDA), Hospital Infantil Sabará, Instituto Pensi, Fundação José Luiz Egydio Setubal, São Paulo, Brazil; 18 Departamento de Psicobiologia da Universidade Federal de São Paulo, São Paulo, Brazil; 19 Instituto del Deporte, Universidad de las Américas, 9170022 Santiago, Chile; 20 Departamento de Pediatria da Universidade Federal de São Paulo, São Paulo, Brazil; 21 Healthy Active Living and Obesity (HALO) Research Group, Children’s Hospital of Eastern Ontario Research Institute, Ottawa, Ontario Canada; 22 School of Epidemiology and Public Health, Faculty of Medicine, University of Ottawa, Ottawa, Ontario Canada

**Keywords:** 24-h movement behaviors, Physical activity, Sedentary time, Sleep duration, Epidemiology

## Abstract

**Background:**

24-hour movement behaviors, including moderate-to-vigorous physical activity (MVPA), sedentary time (ST), and sleep duration, have important implications for health across the lifespan. However, no studies exist that have examined the integration of these 24-hour movement behaviors in Latin America. The purpose of this study was to examine the prevalence of meeting the Canadian 24-Hour Movement Guideline recommendations and sociodemographic correlates of meeting the guidelines in adults from eight Latin American countries.

**Methods:**

This was a multi-national cross-sectional study of 2338 adults aged 18 to 64 years from the Latin American Study of Nutrition and Health. MVPA and ST data were collected using accelerometers. Sleep duration was self-reported using a daily log. Socio-demographic correlates included sex, age, education level, and marital status. Meeting the 24-hour movement guidelines was defined as: ≥150 min/week of MVPA; ≤8 h/day of ST; and between 7 and 9 h/day of sleep. Logistic regression models were estimated on pooled data.

**Results:**

The prevalence of adults who met the MVPA, ST, sleep duration, and integrated recommendations was 48.3, 22.0, 19.4, and 1.6%, respectively. Overall, being a woman (OR: 0.72; 95%CI: 0.55,0.93) and having a middle (0.63; 0.47,0.85) or high education level (0.31; 0.17,0.56) was associated with lower odds of meeting all three of the 24-hour movement guideline recommendations. Being married (1.70; 1.25,2.29) was associated with greater odds of meeting all three recommendations. Being a woman (0.46; 0.39,0.55), aged 50-64 years (0.77; 0.60,0.97), and married (0.79; 0.65,0.96) were associated with lower odds of meeting the MVPA recommendation. Having a middle (0.64; 0.50,0.80) or high (0.36; 0.23,0.55) education level was associated with lower odds and being married (1.86; 1.46,2.36) was associated with greater odds of meeting the ST recommendation. Being a woman (0.63; 0.51,0.78) was associated with lower odds; whereas being aged 50-64 years (1.40; 1.04,1.88) and having a middle education level (1.37; 1.09,1.73) were associated with greater odds of meeting the sleep duration recommendation.

**Conclusions:**

Overall, the proportion of Latin American adults achieving healthy levels of 24-hour movement behaviors was low. Further efforts are needed to promote more MVPA, less ST, and sufficient sleep in Latin American adults.

**Trial registration:**

Clinical Trials NCT02226627. Retrospectively registered on August 27, 2014.

## Background

Engaging in satisfactory levels of physical activity, low levels of sedentary time, and getting sufficient sleep throughout the day is beneficial for health among children, adolescents, and adults [1–4]. Given that a 24-hour period consists of three types of movement behaviors (i.e., physical activity, sedentary behaviors, and sleep) [4], regularly monitoring the levels and patterns of these behaviors is essential for population health measurement and surveillance [5].

The World Health Organization (WHO) [6] and the National Sleep Foundation [7] offer recommendations for physical activity and sleep for different age groups. Although no time-specific benchmark is presented for sedentary time, it is usually advised to reduce time spent being sedentary [4, 6]. Based on the emerging evidence and a better understanding of the importance of considering all of the 24-hour movement behaviors, in an integrated fashion, several countries have developed and released public health guidelines that combine recommendations for physical activity, sedentary behavior, and sleep for children and youth [8–13]. Canada was among the first in the world to develop and release 24-hour movement guidelines for adults aged 18-64 years and adults aged 65 years or older, which included time-specific recommendations for physical activity, sedentary behaviours, and sleep. In doing so, Canada was first country to complete the ‘family’ of 24-hour movement recommendations for all age groups [8]. To date, there is no evidence offered to appreciate the levels of 24-hour movement behaviors among inhabitants from Latin American countries using globally recognized benchmarks such as international physical activity guidelines [6], sleep recommendations [7], or the recently released Canadian 24-Hour movement guidelines. Determining the frequency of individuals meeting these new public health guidelines is crucial to notify future health promotion and disease prevention policies and interventions. However, most previous investigation has been conducted in middle- and high- income countries [14–16].

Latin American countries are among the highest urbanized countries in the world with high population density and pronounced social inequality [17]. Furthermore, Latin America is characterized by high levels of diversity in terms of environment, access to health care, and population aging [18]; these factors inhibit healthy lifestyles, as well as increase the frequency of other risk factors for and prevalence or incidence of non-communicable diseases, such as obesity [19, 20]. Therefore, it is essential to examine the proportion of inhabitants within Latin American countries complying with established 24-hour movement guidelines to inform which percentage of adults engage in healthy levels of 24-hour movement behaviors across the day.

Previous studies have used differents methods to evaluate physical activity, sedentary time, and sleep in Latin America [20–26]. Much of this difference, however, may be elucidated by inconsistencies in the methodologies used to decrease, process, and analyze the data. This limitation can be overcome by combining and reprocessing individual level information from existing research in a harmonized and homogenous manner. This would provide a more consistent and comprehensive estimation of the levels of 24-hour movement behaviors in Latin America populations that could inform public health policy-makers across Latin America.

The Latin American Study of Nutrition and Health (Estudio Latinoamericano de Nutrición y Salud - ELANS) [27] has advanced standardized methods to create equivalent health measures across countries. Results from such a harmonized approach will provide more comparable estimates of 24-hour movement behaviors which can be used to inspire policy makers, governments, and local and national stakeholders to take action to facilitate structural changes designed at promoting increased physical activity, decreased sedentary time, and sufficient sleep duration. Therefore, the aims of this study were to (a) investigate the proportion of adults meeting the individual (moderate-to-vigorous physical activity, sedentary time, and sleep duration) and integrated Canadian 24-Hour Movement Guideline recommendations, and (b) examine sociodemographic correlates of adherence to the individual and integrated guidelines across eight Latin American countries [8]. In this manuscript, we used the Canadian 24-Hour Movement Guideline recommendations because these were the first in the world to provide time-specific recommendations for all three of the 24-hour movement behaviours.

## Methods

### Study design

The data used in the present study were derived from the 2014-2015 cycle of the ELANS (https://es.elansstudy.com/) international database. The ELANS is an observational, epidemiological, cross-sectional study conducted in eight countries in Latin America (Argentina, Brazil, Chile, Colombia, Costa Rica, Ecuador, Peru, and Venezuela), that focuses on urban residents. More details about the methods and design of the ELANS are described elsewhere [27, 28]. The overarching ELANS protocol was approved by the Western Institutional Review Board (#20,140,605) and is registered at ClinicalTrials.gov (#NCT02226627). Ethical approval was obtained from each local institutional review board and participants’ informed consent/assent was obtained.

### Recruitment and participants

Participants were chosen using a random complex, multistage sampling frame with a random selection of Primary Sampling Units (PSUs) and Secondary Sampling Units (SSUs). The PSUs were areas (e.g. counties, municipalities, neighbourhoods, residential areas) within each selected city in each country. An “n” size relative to population weight was used for the selection of PSUs. In this instance, a simple random sampling of “n” with replacement was performed to adhere to the principle of statistical independence of the selection of the areas included in the PSU sample. For these random selections, the probability proportional to size method was applied. Thus, within each of the areas included in the PSU distribution, a representative sample of SSUs was randomly designated using the probability proportional to size method [20, 28].

For the selection of households within SSUs, we implemented a four-stage, systematic randomization by establishing a selection interval: (1) the total urban population was used to proportionally describe the main regions and to select cities representing each region; (2) the sampling points (survey tracts) of each city were randomly designated; (3) clusters of households were selected from each sampling unit; and (4) the designated respondent within each household was selected using the birthday method. In each country, stratified recruitment of participants was completed across sex, age, and socio-economic status. Sample size calculations considered a survey design effect of 1.75 with a maximum sampling error of 3.5% and *p* < 0.05, resulting in a required sample size of 9090. Details have been previously published [20, 28].

In total, 92 cities participated in the ELANS (7 to 23 cities from each country). A total sample of 10,134 people (aged 15.0–65.0 years) were invited to participate in the ELANS study. However, 9218 participants (4809 women) provided valid data and were included in the ELANS study (response rate: 91%). A subsample of 2732 participants aged 15 to 65 years were asked to provide accelerometer data, which represented 29.6% of the total ELANS sample (N = 9218) [29, 30]. The accelerometer subsample were randomly selected to fill quotas by sex, age, and socio-economic status. For logistical and financial reasons, efforts were made to ensure that a range of 23–34% of participants from each specific country each sample wore the accelerometer on all seven days [30, 31].

We excluded adolescents aged 15 to 17 years from the manuscript because the ELANS did not contain adolescents of younger ages. Further, adolescents may have limited independent mobility [32] when compared to adults. In addition, the existing 24-hour movement guideline recommendations are different for adolescents and adults [6, 8]. Furthermore, we also excluded participants aged 65 years based on the age range (i.e., 18-64 years) used in the Canadian guidelines to distinguish adults from older adults [8]. The current manuscript is based on a sample of 2338 participants aged 18 to 64 years with valid accelerometer data representing 25.4% of the total ELANS sample.

For the subsample of participants examined for this secondary data analysis, data was collected via two home visits. At the first visit, the designated respondents received instructions regarding the use of an accelerometer along with a diary to be filled out for seven consecutive days. The second visit which took place eight days later included the administration of the questionnaire and the retrieval of the accelerometer and diary.

### 24-hour movement behaviors

Mean min/day of moderate-to-vigorous physical activity and sedentary time were measured using the GT3X+ Actigraph (Fort Walton Beach, FL, United States), a triaxial accelerometer, that captures acceleration movements in three axes (Vertical, Horizontal, and Perpendicular) [33]. Prior studies have shown the GT3X+ Actigraph to have acceptable technical reliability for physical activity and sedentary behavior [33, 34].

Participants were instructed to wear the accelerometer attached to an elasticized belt at hip level (mid-axillary), from the time they woke up until bed time at night for 7 consecutive days and to remove the accelerometer any time they performed activities that involved the use of water such as bathing or swimming. Days with at least 10 h of recorded wear time were considered valid [35]. A participant was included in the analysis if they had at least five valid days of data, including at least one weekend day. After exclusion of the nocturnal sleep period time, periods with at least 60 min of consecutive zero accelerometer counts were categorized as non-wear time [36]. Details on accelerometer data have been published elsewhere [29, 30].

Data were processed using ActiLife software (V6.0; ActiGraph, Pensacola, FL). Data were collected at a sampling rate of 30 Hz and downloaded in epochs of 60 s [37]. Accelerometer counts were used to classify sedentary time (<100 activity counts/minute) and moderate-to-vigorous physical activity (≥1952 activity counts/minute) [38, 39]. Participants were instructed to complete a daily log, and to report the time they put the accelerometer belt on and the time when it was removed. Sleep duration was calculated by identifying non-wear time during valid accelerometer days, identifying the time between going to bed (removing device) and waking up (wearing the device) [40].

### Sociodemographic characteristics

Sociodemographic correlates were selected a priori based on availability of data from the ELANS study and previous evidence of their associations with physical activity, sedentary time, and sleep in adults [14, 41]. The sociodemographic correlates were assessed using standard questionnaires during face-to-face interviews and included sex (women, men), age (18-34, 35-49, and 50-65 years), education level (low [basic or lower], middle [elementary], and high [university degree]), and marital status (single [not married, widowed, and divorced] or married). Further details can be found in a previous study [27, 29].

### Statistical analysis

Weighting was done according to sociodemographic characteristics, sex, socioeconomic level, and country. Descriptive statistics, including mean, percentage, and 95% confidence intervals (95%CI) were calculated for the sociodemographic correlates and for moderate-to-vigorous physical activity, sedentary time, and sleep duration. Significant differences by sex and country were analyzed by overlapping 95%CI [42].

Each participant was categorized as either “meeting” or “not meeting” the time-specific recommendations outlined within the Canadian 24-Hour Movement Guidelines for adults aged 18-64 years. The recommendations were as follows: (1) engage in at least 150 min/week of moderate-to-vigorous physical activity; (2) spend ≤8 h/day in sedentary time; and (3) obtain between 7 and 9 h/day of sleep. The participants who met all three recommendations for moderate-to-vigorous physical activity, sedentary time, and sleep duration were categorized as meeting the integrated Canadian 24-Hour Movement Guidelines [8]. The proportion of participants meeting the physical activity, sedentary time, and sleep duration recommendations by sex and by country were also calculated.

Logistic regression models (odds ratio: OR; 95%CI) with a binary dependent variable (0 = not meeting the moderate-to-vigorous physical activity, sedentary time and sleep duration recommendations, 1= meeting the moderate-to-vigorous physical activity, sedentary time, and sleep duration recommendations) were performed to analyze the association between sociodemographic correlates and meeting vs. not meeting the individual and integrated guideline recommendations adjusted for city, region, and country. Data analysis was performed using IBM SPSS Statistics 22 (SPSS Inc., IBM Corp., Armonk, New York, NY, USA) with the level of significance set at *p* < 0.05.

## Results

There were no significant differences (*p* > 0.05) between the participants who were asked to wear an accelerometer and those who did not by sex, socioeconomic level, educational level, and marital status. Participant characteristics are presented in Table 1. Overall, 53.4% (95%CI: 51.4, 55.4) of the sample consisted of females and the mean age was 38.2 (95%CI: 37.7, 38.7) years. Overall, 44.7% (95%CI: 42.7, 46.7) of participants were aged <35 years, 56.8% (95%CI: 54.7, 58.8) had low education level, and 52.5% (95%CI: 50.5, 54.5) were married. The country with the highest proportion of participants was Brazil (19.5%; 95%CI: 17.9, 21.1) and the country with the lowest proportion was Costa Rica (9.4%; 95%CI: 8.2, 10.5).

**Table 1:** Participant characteristics (mean or % [95%CI]). 2014-2015 Latin American Study of Nutrition and Health

Characteristics	Mean or % (95% CI) N = 2338
Sex	
Women	53.4 (51.4, 55.4)
Men	46.6 (44.6, 48.6)
Age (years)	38.2 (37.7, 38.7)
Age interval (years)	
18-34	44.7 (42.7, 46.7)
35-49	32.8 (30.9, 34.7)
50-64	22.5 (20.8, 24.2)
Educational Level	
Low	56.8 (54.7, 58.8)
Middle	32.2 (30.3, 34.1)
High	11.0 (9.8, 12.3)
Marital Status	
Single	47.5 (45.3, 49.7)
Married	52.5 (50.5, 54.5)
Country	
Argentina	11.2 (9.9, 12.5)
Brazil	19.5 (17.9, 21.1)
Chile	10.6 (9.3, 11.8)
Colombia	12.4 (11.1, 13.8)
Costa Rica	9.4 (8.2, 10.5)
Ecuador	10.7 (9.4, 11.9)
Peru	12.6 (11.2, 13.9)
Venezuela	13.8. (12.4, 15.2)

Overall, mean values for moderate-to-vigorous physical activity, sedentary time, and sleep duration were 34.4 min/day (95%CI: 33.4, 35.4), 565.1 min/day (95%CI: 560.3, 569.4), and 10.3 h/day (95%CI: 10.2, 10.4), respectively. On average, men accumulated significantly more minutes of moderate-to-vigorous physical activity and sedentary time than women (mean difference: 12.3 and 15.2 min/day); however, no differences were observed in sleep duration between men and women (mean difference: 0.2 h/day) (Table 2).

**Table 2:** Time spent (mean [95%CI]) in moderate-to-vigorous physical activity, sleep duration, and sedentary behavior. 2014-2015 Latin American Study of Nutrition and Health

	MVPA (min/day)	Sedentary time (min/day)	Sleep duration (hours/day)
Overall	34.4 (33.4, 35.4)	565.1 (560.3, 569.4)	10.3 (10.2, 10.4)
Sex			
Women	28.7 (27.6, 29.8)	558.0 (552.1, 563.8)	10.4 (10.3, 10.5)
Men	41.0 (39.2, 42.9)	573.2 (566.2, 580.1)	10.2 (10.1, 10.3)
Country			
Argentina	32.3 (29.5, 35.4)	582.0 (567.0, 596.6)	10.1 (9.8, 10.3)
Brazil	33.4 (31.4, 35.7)	549.4 (539.0, 560.2)	10.6 (10.4, 10.8)
Chile	39.7 (36.9, 42.7)	548.6 (533.9, 561.8)	9.7 (9.5, 10.0)
Colombia	33.8 (31.1, 36.7)	563.8 (552.3, 577.4)	10.8 (10.6, 11.1)
Costa Rica	33.0 (29.6, 37.1)	555.9 (540.8, 570.6)	10.7 (10.4, 10.9)
Ecuador	38.9 (34.9, 42.9)	566.0 (549.8, 580.0)	10.0 (9.7, 10.2)
Peru	35.3 (32.5, 38.5)	592.8 (578.8, 606.5)	9.9 (9.7, 10.2)
Venezuela	30.5 (28.1, 33.0)	568.9 (556.2, 580.5)	10.6 (10.3, 10.8)

*MVPA* moderate-to-vigorous physical activity

Levels of moderate-to-vigorous physical activity were highest in Chile (mean: 39.7 min/day; 95%CI: 36.9, 42.7) and the lowest in Venezuela (mean: 30.5 min/day; 95%CI: 28.1, 33.0) and the mean difference between these two countries was 9.2 min/day. Sedentary time was the highest in Peru (mean: 592.8 min/day; 95%CI: 578.8, 606.5) and lowest in Brazil (mean: 549.4 min/day; 95%CI: 539.0, 560.2), with a mean difference of 43.4 min/day between these two countries. For sleep duration, the mean difference between Colombia (mean: 10.8 h/day; 95%CI: 10.6, 11.1; highest value) and Chile (mean: 9.7 h/day; 95%CI: 9.5, 10.0; lowest value) was 1.1 h/day (Table 2).

**Fig. 1:**
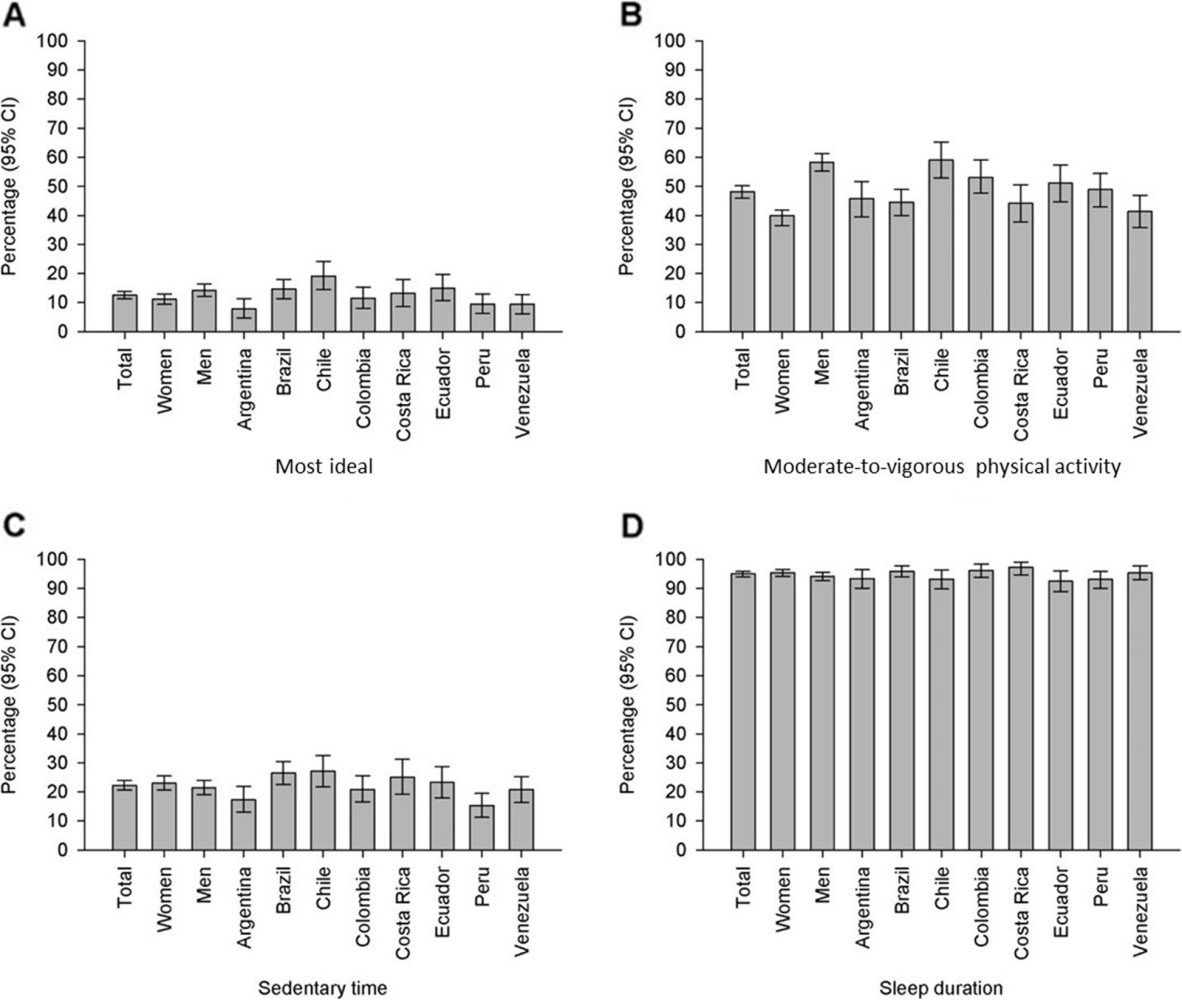
Proportion of participants that met the individual and integrated 24-hour movement guideline recommendations. 2014-2015 Latin American Study of Nutrition and Health

Figure 1 shows the proportion of participants who met all three 24-hour movement guideline recommendations and each of the individual recommendations for moderate-to-vigorous physical activity, sedentary time, and sleep duration. Overall, the proportion of Latin American adults who met all three of the 24-hour movement guideline recommendations was only 1.6% (1.2% in women; 2.1% in men) (Fig. 1 A). A total of 48.3% of participants (39.4% in women; 58.7% in men) met the moderate-to-vigorous physical activity recommendation (Fig. 1B), whereas 22.0% met the sedentary time recommendation (22.7% in women; 21.2% in men) (Fig. 1 C), and 19.4% met the sleep duration recommendation (16.5% in women; 22.8% in men) (Fig. 1D).

Associations between sociodemographic correlates and meeting vs. not meeting the individual (moderate-to-vigorous physical activity, sedentary time, and sleep duration) and integrated recommendations are shown in Table 3. Being a woman (OR: 0.72; 95%CI: 0.55, 0.93), and having a middle (OR: 0.63; 95%CI: 0.47, 0.85) or high education level (OR: 0.31; 95%CI: 0.17, 0.56) were significantly associated with a lower likelihood of meeting the integrated guideline recommendations On the other hand, marital status (married; OR: 1.70; 95%CI: 1.25, 2.29) was significantly associated with a greater likelihood of meeting all three recommendations. Being a woman (OR: 0.46; 95%CI: 0.39, 0.55), aged 50-64 years (OR: 0.77; 95%CI: 0.60, 0.97), and married (OR: 0.79; 95%CI: 0.65, 0.96) were significantly associated with a lower likelihood of meeting the moderate-to-vigorous physical activity recommendation. Middle (OR: 0.64; 95%CI: 0.50, 0.80) and high (OR: 0.36; 95%CI: 0.23, 0.55) education level was significantly associated with a lower likelihood and being married (OR: 1.86; 95%CI: 1.46, 2.36) was significantly associated with a greater likelihood of meeting the sedentary time recommendation. Being a woman (OR: 0.63; 95%CI: 0.51, 0.78) was associated with lower odds and being aged 50-64 years (OR: 1.40; 1.04, 1.88) and having a middle education level (OR: 1.37; 95%CI: 1.09, 1.73) were associated with greater odds of meeting the sleep duration recommendation.

**Table 3:** Logistic regression models [OR (95% CI)] showing associations between sociodemographic correlates and meeting moderate-to-vigorous physical activity, sedentary time, sleep duration, and all three recommendations. 2014-2015 Latin American Study of Nutrition and Health

Sociodemographic correlates	Most ideal OR (95%CI)	MVPA^a^ OR (95%CI)	Sedentary time^b^ OR (95%CI)	Sleep duration^c^ (95%CI)
Sex				
Men	Ref	Ref	Ref	Ref
Women	0.72 (0.55, 0.93)**	0.46 (0.39, 0.55)***	1.05 (0.85, 1.29)	0.63 (0.51, 0.78)*
Age interval (years)				
18-34	Ref	Ref	Ref	Ref
35-49	1.20 (0.88, 1.63)	1.00 (0.81, 1.23)	1.06 (0.83, 1.35)	1.21 (0.93, 1.57)
50-64	0.90 (0.63, 1.29)	0.77 (0.60, 0.97)*	0.92 (0.69, 1.21)	1.40 (1.04, 1.88)*
Educational level				
Low	Ref	Ref	Ref	Ref
Middle	0.63 (0.47, 0.85)**	1.20 (0.99, 1.45)	0.64 (0.50, 0.80)***	1.37 (1.09, 1.73)*
High	0.31 (0.17, 0.56)***	1.13 (0.86, 1.50)	0.36 (0.23, 0.55)***	1.31 (0.93, 1.83)
Marital Status				
Single	Ref	Ref	Ref	Ref
Married	1.70 (1.25, 2.29)**	0.79 (0.65, 0.96)**	1.86 (1.46, 2.36)***	0.86 (0.68, 1.10)

*MVPA* moderate-to-vigorous physical activity; *OR* odds ratio;* 95%CI* 95% confidence interval

Models adjusted for city, region, and country

* *p* < 0.05; ** *p* < 0.01; *** *p* < 0.001;

^a^ 0 = <150 min/week; 1 = ≥150 min/week;

^b^ 0 = ≤8 h/day; 1 = >8 h/day;

^c^ 0 = <7 h/day; 1 = ≥ 7 h/day

## Discussion

The present study was the first to examine the prevalence and sociodemographic correlates of meeting vs. not meeting 24-hour movement guideline recommendations using Latin American data. From these data, we found that only 1.6% of Latin American adults met the integrated 24-hour movement guideline recommendations. However, nearly half of adults met the moderate-to-vigorous physical activity recommendation, and a fifth met the sedentary time and sleep duration recommendations. Being a woman, and having a middle or high education level was associated with lower odds of meeting all three of the 24-hour movement guideline recommendations. Being married was associated with greater odds of meeting all three recommendations of the 24-hour movement guidelines. Being a woman, aged 50-64 years and married were associated with lower odds of meeting the MVPA recommendation. Having a middle or high education level was associated with lower odds and being married was associated with greater odds of meeting the ST recommendation. Being a woman was associated with lower odds; whereas being aged 50-64 years and having a middle education level were associated with greater odds of meeting the sleep duration recommendation.

The evidence presented herein on the proportion of Latin American adults who met the integrated 24-hour movement behavior recommendations (i.e., combination of moderate-to-vigorous physical activity, sedentary time, and sleep recommendations) suggest that adherence to the recommendations, in their entirety, is low (1.6%). Liangruenrom et al. [14] found that around one in five Thai adults met the overall 24-hour movement guidelines in 2015. Another study found that in the same year only 0.4% of Korean adults met a similar ‘most ideal’ combination of movement behaviors [43]. However, a direct comparison between these findings is not possible, as there are differences in methodology used to assess movement behavior and categorise participants. For instance, both studies [14, 43] used different questionnaires to estimate daily physical activity, sedentary time and sleep duration, rather than a 24-hour time-use diary. Further, potential comparisons are also limited due to cultural differences between countries and differences in the recommendations for the specific movement behaviours.

The current findings suggest that sex, education level, as well as marital status are associated with guideline adherence. Several studies have examined correlates associated with meeting the existing movement behavior recommendations, including sex, education level, and marital status [14, 43, 44]. Low adherence to the moderate-to-vigorous physical activity recommendation has been previously reported among women [45]. In particular, Latin American women seem to lag behind in moderate-to-vigorous physical activity compared with men in international studies [22, 29, 45]. Increasing moderate-to-vigorous physical activity levels would be beneficial for the population [3, 46] and would also increase the proportion of inhabitants meeting the integrated 24-hour movement recommendations. Intervention and public health promotion efforts to encourage moderate-to-vigorous physical activity and enhance compliance with the 24-hour movement guideline recommendations are necessary in this population subgroup.

This international study measured intensities and described patterns of moderate-to-vigorous physical activity and sedentary time in eight Latin American countries, using a comparable, reliable and validated device [33]. ELANS is the initial study to account for mean levels of physical activity and sedentary time using nationally-representative samples of urban inhabitants from Latin American countries based on objective methods. We found similar values for time spent in moderate-to-vigorous physical activity in adults from Latin America and high-income countries (34.4 min/day vs. 35.6 min/day) [45]. However, the percentage of adults that met the moderate-to-vigorous physical activity guidelines was lower in Latin America than high-income countries (48.3% vs. 68.2%), respectively [45].

Our results generally support the literature suggesting that education level and marital status are the key correlates of sedentary time among adults in high-income countries [47–49], however, no clear associations between sex and sedentary time appear to exist [49]. In this study, the proportion of the total sample with sedentary time >8 h/day was 78%, which far exceeded the corresponding proportions in specific Latin American countries (Brazil [2.6%], Colombia [5.4%], Argentina [16.3%] using self-reported overall sedentary time reported in a previous international study [50]. The use of objective measures to assess physical activity and sedentary time are more accurate than self-reported data and a study from Latin America found large differences between sedentary time values for self-report and accelerometer measurements [31]. We reinforce the need for additional studies that consider the cut-off point of 8 h/day in sedentary time or investigate different cut-off points to evaluate this behavior with objective methods in Latin America. We found that around one in five Latin American adults met the sedentary time recommendation. 20% of the adults from high-income countries (i.e., England [22%], Norway [11%], Portugal [33%], and Sweden [30%] spent ≤7.5 h/day in sedentary pursuits based on accelerometer data [45]. Development of intervention programs should inform policy and actions to reduce sedentary time among inhabitants from middle (i.e., Latin America countries) and high-income countries.

The average sleep duration was 10.3 h/day in our adult sample, which far exceeds the corresponding averages in low and middle-income countries [51]. A systematic review and meta-analysis showed mean 7.6 h/day of self-reported sleep duration in adults and older adults using data from 17 studies from these countries [51]. Self-report measures of sleep duration may considerably underestimate sleep time, when compared with objective measures [52]. Similar with other epidemiological studies, we found that sex, age, and education level were significantly associated with meeting vs. not meeting the sleep duration recommendation, such that middle education level were more likely to meet this recommendation [53]. Previous literature have demonstrated the benefits of sufficient sleep, both separately and in combinations with physical activity and sedentary time for health (i.e., adiposity level, HDL-cholesterol, and triglycerides) [54]. Given the links with poor health, the increasing prevalence of long sleep duration may be an early marker of declining public health [55]. Given the aforementioned, it is important to identify modifiable factors associated with sleep problems, which can be targeted via public prevention programs.

Latin American adults who were married were significantly less likely to meet the moderate-to-vigorous physical activity recommendation, but significantly more likely to meet the sedentary time recommendation compared to those who were single. In line with the findings of our analyses, Liangruenrom et al. reported a positive association (OR: 1.44; 95%IC: 1.37, 1.51) of married marital status with sedentary time recommendations (defined as interrupting sedentary time every 2 h) compared with never married marital status [14]. Considering the negative health effects of prolonged sedentary time and a low physical activity level [1, 3], it should be highlighted that married individuals may benefit from interventions aiming to decrease sedentary time and increase moderate-to-vigorous physical activity.

Reliable and valid data on daily movement behaviors are needed in order to enhance the understanding of dose-response relationships between movement behaviors and various health outcomes. Additionally, future research should consider the implementation of behavior-change interventions and counseling strategies to facilitate a sustainable healthy and active lifestyle. Such research could also provide viable information for the development of evidence-based 24-hour movement recommendations that include strategies to achieve them. At this time, there is, however, limited data on 24-hour movement behaviors in Latin American countries along with recommendations for strategies to enhance an active and healthy lifestyle. Such information is a critical contributor for strategic decisions regarding health policy, practice and overall health promotion among Latin American adults. Given the impact of movement behavior across the entire lifespan the promotion of physical activity is key to future public health.

This study was not without its limitations, including the cross-sectional design, which prevents conclusions regarding causality from being made. The temporality of when the data were collected (i.e., 2014-2015) relative to the release of the 24-Hour Movement Guidelines (i.e., 2020) is a limitation. It is possible that the movement behaviors of adult populations across Latin America have changed over this time period. Another potential limitation was that sleep duration was derived from non-wear time of valid days. Although participants were instructed to remove the device only to sleep and took notes in their daily logs, and these were matched to identify potential problems, there was no objective measure of sleep available. Furthermore, the ELANS study did not assess recreational screen time as suggested by the Canadian 24-Hour Movement Guidelines. There are no specific public health recommendations concerning 24-Hour Movement Guidelines for inhabitants from Latin America. On the other hand, there are several strengths of the present study. First, to the authors’ knowledge, this was the first study to describe the levels and sociodemographic correlates of adherence to 24-hour movement guideline recommendations among Latin American adults. Second, the large sample size from eight Latin American countries using a standardized methodology across a consortium of participating countries was a strength. Third, objective measures were used to assess moderate-to-vigorous physical activity and sedentary time, which is rare in Latin American countries where the majority of previous research has relied on self-reported instruments [29, 31].

## Conclusions

A large majority of Latin American adults do not meet the integrated 24-hour movement guideline recommendations. Further actions are needed to promote more moderate-to-vigorous physical activity, less sedentary time, and satisfactory sleep duration in Latin American adults from urban areas. Future studies should explore prevalence and correlates of moderate-to-vigorous physical activity, sedentary time, and sleep patterns (e.g., duration, quality, consistency) in Latin American inhabitants in greater detail. Moreover, currently 24-hour movement guideline recommendations do not exist in Latin America; it is important for countries to develop these to allow for greater measurement, surveillance and promotion of movement behaviors among Latin American adults. Our findings indicate that regional public health efforts are needed to promote more physical activity, less sedentary time, and adequate sleep among Latin American adults to increase the proportion of individuals meeting existing 24-hour movement guideline recommendations.

## Data Availability

The datasets generated and/or analyzed during this study are not publicly available due the terms of consent/assent to which the participants agreed but are available from the corresponding author upon reasonable request. Please contact the corresponding author to discuss availability of data and materials.

## References

[1] Rezende LFM, Lee DH, Ferrari G, Giovannucci E (2020). Confounding due to pre-existing diseases in epidemiologic studies on sedentary behavior and all-cause mortality: a meta-epidemiologic study. Ann Epidemiol.

[2] Watson NF, Badr MS, Belenky G, Bliwise DL, Buxton OM, Buysse D (2015). Recommended Amount of Sleep for a Healthy Adult: A Joint Consensus Statement of the American Academy of Sleep Medicine and Sleep Research Society. Sleep.

[3] Wang Y, Nie J, Ferrari G, Rey-Lopez JP, Rezende LFM (2021). Association of Physical Activity Intensity With Mortality: A National Cohort Study of 403681 US Adults. JAMA Intern Med.

[4] Tremblay MS, Aubert S, Barnes JD, Saunders TJ, Carson V, Latimer-Cheung AE (2017). Sedentary Behavior Research Network (SBRN) - Terminology Consensus Project process and outcome. Int J Behav Nutr Phys Act.

[5] Bauman AE, Reis RS, Sallis JF, Wells JC, Loos RJ, Martin BW (2012). Correlates of physical activity: why are some people physically active and others not?. Lancet.

[6] Bull FC, Al-Ansari SS, Biddle S, Borodulin K, Buman MP, Cardon G (2020). World Health Organization 2020 guidelines on physical activity and sedentary behaviour. Br J Sports Med.

[7] Hirshkowitz M, Whiton K, Albert SM, Alessi C, Bruni O, DonCarlos L (2015). National Sleep Foundation’s sleep time duration recommendations: methodology and results summary. Sleep Health.

[8] Ross R, Chaput JP, Giangregorio LM, Janssen I, Saunders TJ, Kho ME (2020). Canadian 24-Hour Movement Guidelines for Adults aged 18-64 years and Adults aged 65 years or older: an integration of physical activity, sedentary behaviour, and sleep. Appl Physiol Nutr Metab.

[9] Okely AD, Ghersi D, Hesketh KD, Santos R, Loughran SP, Cliff DP (2017). A collaborative approach to adopting/adapting guidelines - The Australian 24-Hour Movement Guidelines for the early years (Birth to 5 years): an integration of physical activity, sedentary behavior, and sleep. BMC Public Health.

[10] Reilly JJ, Hughes AR, Janssen X, Hesketh KR, Livingstone S, Hill C (2020). GRADE-ADOLOPMENT Process to Develop 24-Hour Movement Behavior Recommendations and Physical Activity Guidelines for the Under 5s in the United Kingdom, 2019. J Phys Act Health.

[11] New Zealand Ministry of Health (2017). Sit less, move more, sleep well: physical activity guidelines for children and young people.

[12] Tremblay MS, Carson V, Chaput JP, Connor Gorber S, Dinh T, Duggan M (2016). Canadian 24-Hour Movement Guidelines for Children and Youth: An Integration of Physical Activity, Sedentary Behaviour, and Sleep. Appl Physiol Nutr Metab.

[13] Tremblay MS, Chaput JP, Adamo KB, Aubert S, Barnes JD, Choquette L (2017). Canadian 24-Hour Movement Guidelines for the Early Years (0-4 years): An Integration of Physical Activity, Sedentary Behaviour, and Sleep. BMC Public Health.

[14] Liangruenrom N, Dumuid D, Craike M, Biddle SJH, Pedisic Z (2020). Trends and correlates of meeting 24-hour movement guidelines: a 15-year study among 167,577 Thai adults. Int J Behav Nutr Phys Act.

[15] Weatherson KA, Joopally H, Wunderlich K, Kwan MY, Tomasone JR, Faulkner G (2021). Post-secondary students’ adherence to the Canadian 24-Hour Movement Guidelines for Adults: Results from the first deployment of the Canadian Campus Wellbeing Survey (CCWS). Health Promot Chronic Dis Prev Can.

[16] McGregor DE, Carson V, Palarea-Albaladejo J, Dall PM, Tremblay MS, Chastin SFM. Compositional Analysis of the Associations between 24-h Movement Behaviours and Health Indicators among Adults and Older Adults from the Canadian Health Measure Survey. Int J Environ Res Public Health. 2018;15(8):1779 10.3390/ijerph15081779 PMC6121426 30126215

[17] United Nations. World Population Prospects: 2019 Revision. New York, NY: United Nations; 2019. (Department of Economic and Social Affairs, Population Division). 2019.

[18] Kruk ME, Gage AD, Joseph NT, Danaei G, Garcia-Saiso S, Salomon JA (2018). Mortality due to low-quality health systems in the universal health coverage era: a systematic analysis of amenable deaths in 137 countries. Lancet.

[19] Collaboration NCDRF (2017). Worldwide trends in body-mass index, underweight, overweight, and obesity from 1975 to 2016: a pooled analysis of 2416 population-based measurement studies in 128.9 million children, adolescents, and adults. Lancet.

[20] Ferrari G, Werneck AO, Silva DR, Kovalskys I, Gomez G, Rigotti A, et al. Agreement Between Self-Reported and Device-Based Sedentary Time among Eight Countries: Findings from the ELANS. Prev Sci. 2021. 10.1007/s11121-021-01206-x 33502675

[21] Werneck AO, Baldew SS, Miranda JJ, Diaz Arnesto O, Stubbs B, Silva DR (2019). Physical activity and sedentary behavior patterns and sociodemographic correlates in 116,982 adults from six South American countries: the South American physical activity and sedentary behavior network (SAPASEN). Int J Behav Nutr Phys Act.

[22] Werneck AO, Sadarangani KP, Ramirez-Velez R, Baldew SS, Gomes TN, Ferrari G (2020). Macroeconomic, demographic and human developmental correlates of physical activity and sitting time among South American adults. Int J Behav Nutr Phys Act.

[23] Figueiredo TKF, Aguiar RG, Florindo AA, Alves M, Barros MBA, Goldbaum M (2021). Changes in total physical activity, leisure and commuting in the largest city in Latin America, 2003-2015. Rev Bras Epidemiol.

[24] Gajardo YZ, Ramos JN, Muraro AP, Moreira NF, Ferreira MG, Rodrigues PRM (2021). [Sleep-related problems and associated factors among the Brazilian population: National Health Survey, 2013]. Cien Saude Colet.

[25] Troncoso C, Petermann-Rocha F, Brown R, Leiva AM, Martinez MA, Diaz-Martinez X (2018). Patterns of healthy lifestyle behaviours in older adults: Findings from the Chilean National Health Survey 2009-2010. Exp Gerontol.

[26] Matuzaki L, Santos-Silva R, Marqueze EC, de Castro Moreno CR, Tufik S, Bittencourt L (2014). Temporal sleep patterns in adults using actigraph. Sleep Sci.

[27] Fisberg M, Kovalskys I, Gomez G, Rigotti A, Cortes LY, Herrera-Cuenca M (2016). Latin American Study of Nutrition and Health (ELANS): rationale and study design. BMC Public Health.

[28] Ferrari G, Werneck AO, da Silva DR, Kovalskys I, Gomez G, Rigotti A (2020). Is the perceived neighborhood built environment associated with domain-specific physical activity in Latin American adults? An eight-country observational study. Int J Behav Nutr Phys Act.

[29] Ferrari GLM, Kovalskys I, Fisberg M, Gomez G, Rigotti A, Sanabria LYC (2020). Socio-demographic patterning of objectively measured physical activity and sedentary behaviours in eight Latin American countries: Findings from the ELANS study. Eur J Sport Sci.

[30] Ferrari GLM, Kovalskys I, Fisberg M, Gómez G, Rigotti A, Sanabria LYC, et al. Methodological design for the assessment of physical activity and sedentary time in eight Latin American countries - the ELANS study. MethodsX. 2020;S2214-1405(19)30076-3. 10.1016/j.mex.2020.100843 PMC7082600 32211304

[31] Ferrari GLM, Kovalskys I, Fisberg M, Gomez G, Rigotti A, Sanabria LYC (2020). Comparison of self-report versus accelerometer - measured physical activity and sedentary behaviors and their association with body composition in Latin American countries. PLoS One.

[32] Guthold R, Stevens GA, Riley LM, Bull FC (2020). Global trends in insufficient physical activity among adolescents: a pooled analysis of 298 population-based surveys with 1.6 million participants. Lancet Child Adolesc Health.

[33] Sasaki JE, John D, Freedson PS (2011). Validation and comparison of ActiGraph activity monitors. J Sci Med Sport.

[34] Yano S, Koohsari MJ, Shibata A, Ishii K, Frehlich L, McCormack GR, et al. Physical Activity and Sedentary Behavior Assessment: A Laboratory-Based Evaluation of Agreement between Commonly Used ActiGraph and Omron Accelerometers. Int J Environ Res Public Health. 2019;16(17):3126 10.3390/ijerph16173126 PMC6747086 31466248

[35] Brond JC, Arvidsson D (2016). Sampling frequency affects the processing of Actigraph raw acceleration data to activity counts. J Appl Physiol (1985).

[36] Troiano RP, Berrigan D, Dodd KW, Masse LC, Tilert T, McDowell M (2008). Physical activity in the United States measured by accelerometer. Med Sci Sports Exerc.

[37] Colley R, Connor Gorber S, Tremblay MS (2010). Quality control and data reduction procedures for accelerometry-derived measures of physical activity. Health Rep.

[38] Matthews CE, Chen KY, Freedson PS, Buchowski MS, Beech BM, Pate RR (2008). Amount of time spent in sedentary behaviors in the United States, 2003-2004. Am J Epidemiol.

[39] Freedson PS, Melanson E, Sirard J (1998). Calibration of the Computer Science and Applications, Inc. accelerometer. Med Sci Sports Exerc.

[40] Urbanek JK, Spira AP, Di J, Leroux A, Crainiceanu C, Zipunnikov V (2018). Epidemiology of objectively measured bedtime and chronotype in US adolescents and adults: NHANES 2003-2006. Chronobiol Int.

[41] Lee EY, Khan A, Uddin R, Lim E, George L. Six-year trends and intersectional correlates of meeting 24-Hour Movement Guidelines among South Korean adolescents: Korea Youth Risk Behavior Surveys, 2013-2018. J Sport Health Sci. 2020. 10.1016/j.jshs.2020.11.001 PMC10105012 33188965

[42] Lee S, Davis WW, Nguyen HA, McNeel TS, Brick JM, Flores-Cervantes I. Examining Trends and Averages Using Combined Cross-Sectional Survey Data from Multiple Years. CHIS Methodology Paper 2007:1-24.

[43] Lee EY, Carson V, Jeon JY, Spence JC, Tremblay MS (2019). Levels and correlates of 24-hour movement behaviors among South Koreans: Results from the Korea National Health and Nutrition Examination Surveys, 2014 and 2015. J Sport Health Sci.

[44] Knell G, Durand CP, Kohl HW, 3rd, Wu IHC, Pettee Gabriel K. Prevalence and Likelihood of Meeting Sleep, Physical Activity, and Screen-Time Guidelines Among US Youth. JAMA Pediatr. 2019;173(4):387–9. 10.1001/jamapediatrics.2018.4847 PMC6450269 30715096

[45] Loyen A, Clarke-Cornwell AM, Anderssen SA, Hagstromer M, Sardinha LB, Sundquist K (2017). Sedentary Time and Physical Activity Surveillance Through Accelerometer Pooling in Four European Countries. Sports Med.

[46] Rezende LFM, Murata E, Giannichi B, Tomita LY, Wagner GA, Sanchez ZM (2020). Cancer cases and deaths attributable to lifestyle risk factors in Chile. BMC Cancer.

[47] van der Berg JD, Bosma H, Caserotti P, Eiriksdottir G, Arnardottir NY, Martin KR (2014). Midlife determinants associated with sedentary behavior in old age. Med Sci Sports Exerc.

[48] Muller AM, Chen B, Wang NX, Whitton C, Direito A, Petrunoff N (2020). Correlates of sedentary behaviour in Asian adults: A systematic review. Obes Rev.

[49] Prince SA, Roberts KC, Melvin A, Butler GP, Thompson W (2020). Gender and education differences in sedentary behaviour in Canada: an analysis of national cross-sectional surveys. BMC Public Health.

[50] Bauman A, Ainsworth BE, Sallis JF, Hagstromer M, Craig CL, Bull FC (2011). The descriptive epidemiology of sitting. A 20-country comparison using the International Physical Activity Questionnaire (IPAQ). Am J Prev Med.

[51] Simonelli G, Marshall NS, Grillakis A, Miller CB, Hoyos CM, Glozier N (2018). Sleep health epidemiology in low and middle-income countries: a systematic review and meta-analysis of the prevalence of poor sleep quality and sleep duration. Sleep Health.

[52] Schokman A, Bin YS, Simonelli G, Pye J, Morris R, Sumathipala A (2018). Agreement between subjective and objective measures of sleep duration in a low-middle income country setting. Sleep Health.

[53] Mazzotti DR, Guindalini C, Sosa AL, Ferri CP, Tufik S (2012). Prevalence and correlates for sleep complaints in older adults in low and middle income countries: a 10/66 Dementia Research Group study. Sleep Med.

[54] Rollo S, Antsygina O, Tremblay MS (2020). The whole day matters: Understanding 24-hour movement guideline adherence and relationships with health indicators across the lifespan. J Sport Health Sci.

[55] Bin YS, Marshall NS, Glozier N (2013). Sleeping at the limits: the changing prevalence of short and long sleep durations in 10 countries. Am J Epidemiol.

